# NFE2 and PF4 as biomarkers for BET inhibition-induced thrombocytopenia in preclinical and clinical studies

**DOI:** 10.3389/fmed.2025.1592693

**Published:** 2025-08-27

**Authors:** Cindy Zhang, Ke Xu, Faye Wang, Jennifer Price, Julie Panzica, Shodeinde Coker, Oriana Esposito, Danielle Greenawalt, Richard Westhouse, Karen Augustine-Rauch

**Affiliations:** ^1^Department of Discovery Toxicology, Bristol Myers Squibb, Princeton, NJ, United States; ^2^Department of Translational Informatics and Predictive Sciences, Bristol Myers Squibb, Princeton, NJ, United States; ^3^Department of Early Clinical Science, Bristol Myers Squibb, Princeton, NJ, United States

**Keywords:** translational biomarkers, predictive biomarkers, thrombocytopenia, BET inhibition, biomarkers, GATA1, Nfe2, PA4

## Abstract

**Introduction:**

Bromodomain and Extraterminal (BET) proteins play a crucial role in cellular proliferation and differentiation through the epigenetic regulation of gene transcription. As a result, inhibiting BET family proteins emerges as a promising epigenetic approach for treating various cancers. However, clinical trials have indicated that thrombocytopenia is a dose-limiting toxicity associated with BET inhibition. This study aims to explore the mechanism and clinical pharmacology of BMS-986158-induced thrombocytopenia and to identify biomarkers as tools to identify patients at higher risk, thereby better managing toxicity and improving efficacy.

**Methods:**

Blood samples from preclinical rats and clinical trial patients treated with BMS-986158 were collected for transcriptional expression profiling. Target engagement was confirmed by measuring HEXIM1 and monitoring thrombocytopenia following BET inhibition. Genes regulated by GATA1 and associated with thrombopoiesis, including NFE2 and PF4, were investigated. The outcomes of the rat and human studies were compared to identify biomarkers for the early prediction of thrombocytopenia associated with BET inhibition.

**Results:**

Target engagement was confirmed with dose-dependent responses of HEXIM1 expression and platelet counts. Blood samples from rats treated with BMS-986158 showed dose-dependent downregulation of GATA1, NFE2, and PF4 at 24 h or earlier post-treatment. Similarly, patients’ blood samples collected within 24 h post-treatment with BMS-986158 also showed dose-dependent downregulation of GATA1 and PF4 in all treated groups. Significant downregulation of PF4 and NFE2 genes was found in patients with low platelet counts. A strong correlation between the expression of GATA1 and the genes NFE2 and PF4 was observed in both preclinical and clinical studies.

**Discussion:**

The consistent downregulation of GATA1, NFE2, and PF4 transcription within hours post-BMS-986158 treatment in both preclinical and clinical studies demonstrates that BET inhibitors induce thrombocytopenia by altering GATA1 gene expression and its downstream genes, NFE2 and PF4, which regulate megakaryopoiesis and thrombopoiesis. Early detection of transcriptional changes in blood samples during treatment courses positions NFE2 and PF4 as promising biomarkers for proactively monitoring and mitigating treatment-emergent thrombocytopenia.

## Graphical abstract

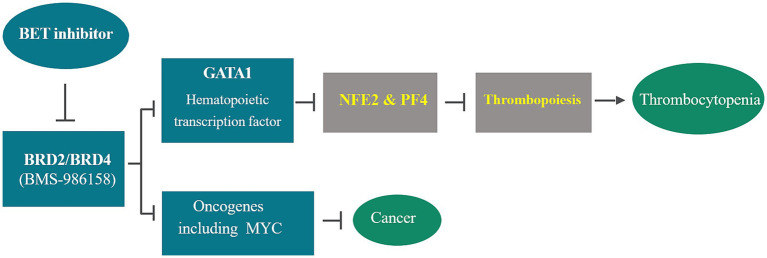

## 1 Introduction

Bromodomain and extraterminal (BET) inhibitors have been developed as anti-cancer drugs due to their roles in cell cycle regulation and RNA polymerase II (Pol II) transcription, including initiating transcription of proto-oncogenes including c-MYC (1–3). Therefore, inhibition of BET family proteins, including BRD2, BRD3, BRD4, and BRDT, represents a promising epigenetic approach for various cancer treatments. Within the BET family, BRD2 selectively interacts with acetylated lysine 12 on histone H4 and transcription factors E2F, which are involved in the cell cycle and DNA synthesis (4). BRD4 plays important roles in cell growth, cell cycle, and DNA replication. It enhances RNA polymerase II C-terminal domain phosphorylation and is a positive regulatory component of P-TEFb (positive transcription elongation factor) (5, 6). BRD4 has been associated with the development of multiple solid tumors (7–9). In addition, BRD3 and BRD4 can be fused with NUT, a nuclear protein in testis, to form an oncoprotein that causes NUT midline carcinoma (10, 11).

BMS-986158, a highly selective and potent inhibitor of BRD2/BRD4, has demonstrated anti-cancer activity through the downregulation of BRD4 and c-MYC expressions in preclinical efficacy studies and a clinical trial for patients with solid tumors (NCT02419417) (12, 13). It has shown robust cytotoxicity and antitumor activity across a broad range of cancer cell lines and tumor models (14, 15).

However, little is known about the consequence of BET inhibition on normal stem cell renewal processes, such as hematopoiesis. Since the report of the first BET pan inhibitor, JQ1, in 2010 (16), more than a dozen BET inhibitors, including BMS-986158, have been developed. Approximately 50 clinical trials have been conducted to evaluate the efficacy, toxicity, and tolerability of BET inhibitors for various cancer treatments.^1^ The outcome of these clinical trials indicated that thrombocytopenia induced by BET inhibition is a primary factor of dose-limiting toxicity (DLT) with BET inhibitor treatment in preclinical toxicity studies and clinical trials (12, 13, 17, 18).

For example, in a recent phase I/II clinical trial on molibresib (GSK525762) for the treatment of relapsed/refractory hematologic malignancies (NCT01943851), among 111 patients, 6 achieved complete responses and 7 achieved partial responses, totaling 13% response rate. However, the most common grade 3 + adverse event was thrombocytopenia, occurring in 37% of patients (19). Similarly, in a first-in-human study of mivebresib (ABBV-075) for patients with relapsed/refractory solid tumors, thrombocytopenia was one of the most common treatment-emergent adverse events (TEAE), presenting in 48% of patients (20). In a phase 1/2a clinical study (NCT02419417), BMS-986158 was evaluated for its safety, tolerability, pharmacokinetics (PK), and pharmacodynamics. Among 83 patients enrolled, 39% developed thrombocytopenia, one of the most treatment-associated side effects.

The objective of this study was to investigate the impact of BET inhibition on a pathway that regulates thrombopoiesis and to explore biomarkers associated with thrombocytopenia following BET inhibitor (BETi) treatment.

It is known that BET inhibition interferes with the normal chromatin occupancy of hematopoietic transcription factor GATA1, subsequently affecting GATA1-mediated transcriptional activation (21). This disruption is believed to impact the downstream target genes regulated by GATA1 and their associated biological functions in hematopoietic cell development, such as megakaryocyte, erythroid maturation and development (22–24), as well as reprogramming of hematopoietic precursors (25, 26).

Therefore, this study aimed to discover transcriptional biomarkers within the GATA1 regulated pathway of thrombopoiesis. The results from both preclinical studies in rats and a human clinical trial with BMS-986158 treatment revealed that Nuclear Factor Erythroid 2 (NFE2) and Platelet Factor 4 (PF4, CXCL4) genes are desirable biomarkers for predicting the risk of thrombocytopenia following BET inhibition. These findings provide a crucial step forward in proactively managing and mitigating thrombocytopenia in patients undergoing BET inhibitor therapy.

## 2 Materials and methods

### 2.1 Preclinical study

#### 2.1.1 BMS-986158 treatment *in vivo* in rats

Sprague Dawley rats (Crl: CD SD-Sprague Dawley, Supplier: Charles River Laboratories, Inc., RRID: SCR_003792) were used in this study. The rats were approximately 8 weeks old and weigh 250-350 g fed with certified 18% Protein Rodent Diet (Harlan Diet #2018C).

Male rats were used in this study based on findings from a preliminary pharmacokinetics study, which showed that female rats exhibited twice the exposure levels and were less tolerant (data not shown). BMS-986158 was administered daily to Sprague Dawley rats (RRID: RGD_70508) via oral gavage at doses of 0, 1, and 5 mg/kg/day for 4 days. The vehicle control consisted of 10% ethanol, 10% TPGS, and 80% PEG300. Each group included 8 rats. On day 5, bone marrow and peripheral blood samples were collected from 5 rats per group. Given the exploratory nature of this study, a formal power calculation was not required. However, the number of animals was the minimum necessary to meet general scientific principles. This study did not constitute an unnecessary duplication of data concerning species, strain, test article, route of administration, or treatment duration.

Animal selection, identification, randomization, husbandry, record keeping, statistical analysis, and record maintenance were conducted in accordance with Standard Operating Procedures and the relevant Institutional Animal Care and Use Committee (IACUC) Standard Protocol and policies. Contaminants known or reasonably anticipated to be present in the diet or drinking water were monitored to ensure they did not reach levels that could compromise the scientific integrity of the study.

#### 2.1.2 Platelet counts

Blood samples were collected from each treatment group of rats, and platelet counts were performed using the Advia 120 Hematology system.

#### 2.1.3 Rat bone marrow cell collection

Rat bone marrow cells were flushed out from femurs using Iscove’s Modified Dulbecco’s Medium (IMDM) culture medium (Gibco™, Cat. #12440053) containing 20% fetal bovine serum (ThermoFisher Scientific, Cat. #A5670701). The cells were then filtered through a 40 μm cell strainer. The collected bone marrow cells were used for the evaluations of megakaryocytes by flow cytometry, megakaryocyte progenitors by Colony Forming Unit (CFU) Assay, and transcription profiling by Real Time PCR (RT-PCR) Assay.

#### 2.1.4 Evaluation of rat megakaryocyte in bone marrow by using flow cytometry

Rat megakaryocyte levels in bone marrow were evaluated by using flow cytometric assay. Collected bone marrow cells were washed with staining buffer (BD Biosciences, Cat. # 554656). Red blood cells were lysed using ACK lysing buffer (Life Technologies, Cat# A10492-01) for approximately 15 min and the lysis reaction was stopped by adding staining buffer to the cells.

Bone marrow cells were stained with Fc block (BD Biosciences, Cat. #550271, RRID: AB_393568) to reduce non-specific binding background, followed by antibody staining with appropriate amounts determined after antibody titration. The antibody cocktail included mouse anti-rat pan leukocyte marker CD45-eF450 (ThermoFisher Scientific, Cat. #48–0461-82, RRID: AB_2574019), bone marrow MK marker CD61-BV480 (BD Biosciences, Cat. #746699, RRID: AB_2743966), erythroid marker anti-erythroid-APC (BD Biosciences, Cat. #562348, RRID: AB_11152955), and transferrin receptor marker on proliferating erythroid cells CD71-PE (BD Biosciences, Cat. #554891, RRID: AB_2650143). A Fixable Viability Dye eFluor™ 780 (eBioscience, Cat. #65–0865-14) was used prior to antibody staining and cell fixation to exclude non-viable cells. Antibody-stained megakaryocytes (CD45 positive / CD61 positive / erythroid negative), with and without treatment of the BET inhibitor BMS-986158, were evaluated using a BD FACSCanto II flow cytometer. Data graphing and statistical analysis were performed using Prism GraphPad (GraphPad Prism, RRID: SCR_002798).

#### 2.1.5 Assessment of megakaryocyte progenitors in bone marrow by using colony forming unit assay

The progenitors of megakaryocytes (MK) in rat bone marrow were evaluated using a CFU assay. CFU-MK colonies, representing the progenitors of MK cells, were counted after bone marrow cells were cultured using MegaCult™-C assay (Stemcell Technologies, Vancouver, Canada, Cat. #04900) with modifications based on Evstatiev’s method (27). The MegaCult™ medium was supplemented with 50 ng/mL recombinant human (rh) TPO, 10 ng/mL recombinant rat (rr) IL-3, 10 ng/mL rrIL-6, and 50 ng/mL rhIL-11 (R&D Systems, Minneapolis, MN, Cat. # 288-TP/CF, Cat. # 2524-RL-025, Cat. # 506-RL-010, and Cat. # 218-IL-005).

Collected bone marrow cells were placed on ice, counted, and diluted to a final concentration of (2.2×10^6) cells/mL in the culture medium. Collagen (Stemcell Technologies, Vancouver, Canada, Cat. # 04902) and the cell suspension were added to the modified MegaCult™ media and transferred to double chamber slides to achieve (1×10^5) cells/slide. All dose groups were run in quadruplicate. The cells were cultured for 7 days, then dehydrated, fixed, and stained according to the manufacturer’s instructions for the MegaCult™-C assay. Megakaryocytic colonies, identified by their brown granular appearance, were counted using a Nikon inverted microscope.

#### 2.1.6 Transcription profiling from rat blood and bone marrow cells

RNA Extraction: 0.1 mL of blood (with EDTA anticoagulant) from each rat was collected into Qiagen’s RNAprotect tube with RNA stabilization buffer (Qiagen, Cat. # 76544) on day 4 after dosing, at 1, 3, 6, and 24 h (on day 5), and on day 15 for transcriptional profiling. RNA was extracted from the blood using Qiagen’s RNeasy® Protect Animal Blood Kit (Qiagen, Cat. # 73224) according to the manufacturer’s recommendations. Collected bone marrow cells were washed with Dulbecco’s Phosphate Buffered Saline (MilliporeSigma, Cat. # D8537, PBS), and 5 million cells were pelleted and stored with 100 μL of RNALater (Qiagen, Cat. # 76140). Total RNA was extracted from the bone marrow sample using the PureLink™ RNA Mini Kit (Thermo Fisher, Cat. # 12183018A) according to the manufacturer’s recommendations.

RT-PCR Assay: RNA samples (~200 ng from blood and 100 ng from bone marrow) were reverse transcribed using the SuperScript™ VILO™ cDNA Synthesis Kit (Thermo Fisher, Cat. # 11754250). After a 1.75x dilution with water, 2 μL of the RT product was used in 20 μL RT-PCR reactions carried out in 384-well plates with the QuantStudio™ 7 Pro Real-Time PCR System (ThermoFisher Scientific). Relative quantitation was calculated using the Delta Delta Ct method: data were first normalized by ACTB and then calibrated by the vehicle control for each time point of the experiment. TaqMan pre-designed primer/probe sets are listed in Table 1.

**Table 1:** TaqMan pre-designed primer/probe sets.

Symbols	Genes	Life technologies assay ID
GATA1	Gata1 (GATA binding protein 1)	Rn00562745_m1
HEXIM 1	HEXIM 1 (Hexamethylene Bisacetamide-Inducible Protein 1)	Rn01466932_s1
PF4	PF4 (platelet factor 4)	Rn01768297_g1
NFE2	NFE2 (nuclear factor, erythroid 2)	Rn01533343_m1
ACTB	Beta-actin (housekeeping gene standard)	Rn00667869_m1

### 2.2 Clinical study design and blood sample collections

Samples for exploratory analysis of biomarkers were collected from 42 patients enrolled in the CA011-011 (NCT02419417) study of BMS-986152, which included subjects with select advanced cancers, such as ovarian cancer, small cell lung cancer, triple negative breast cancer, NUT midline carcinoma, and prostate cancer. The study included three dosing schedules (A, B, and C) and multiple treatment cycles.

Each of the three schedules began with a single dose on Day 1 of Cycle 1 as a ‘run-in’ cycle to assess single-dose safety and pharmacokinetics, followed by a 5–7-day break before initiating Cycle 2. In Schedule A, Cycle 2 followed a 7-day regimen consisting of 5 days on and 2 days off. In Schedules B and C, Cycle 2 followed a 21-day treatment schedule, with Schedule B administering 4 days on and 7 days off, and Schedule C administering 7 days on and 14 days off. The total number of treatment cycles was 9 for Schedule A, 24 for Schedule B, and 19 for Schedule C.

On day 1 of Cycle 1, BMS-986158 was administered as a single oral dose, to assess pharmacokinetic and pharmacodynamic changes at different single oral dose levels. In Cycle 2 and the rest treatment cycles, BMS-986158 was administered once daily (QD) with 5 days on and 2 days off for schedule A, 14 days on and 7 days off for schedule B, and 7 days on and 14 days off for schedule C. The doses were 0.75, 1.25, 2.0, 3.0, or 4.5 mg (13). Longitudinal blood samples available for RNA sequencing (RNAseq) analysis were collected from 32 patients on Schedule A, 4 patients on Schedule B, and 6 patients on Schedule C. Samples were taken at baseline (prior to Cycle 1 treatment), 8 h after the first dose on day 1 of Cycle 2 (Schedule A), 8 h after the end of Cycle 2 on day 14 (Schedule B), and 8 h after the end of Cycle 2 on day 7 (Schedule C).

Additionally, multiple platelet counts were recorded weekly from pre-dose through the end of the treatment cycles. Counts from Day 1 to Day 23 were utilized for analysis and graphing. The clinical sample collection schedules, covering both platelet count and gene expression assessments, are shown below.

The clinical treatment schedule and sample collection timeline are illustrated in Supplementary Figure 1.

### 2.3 Data analysis and statistical tests

Whole transcriptome expression profiling was performed on the collected blood samples using RNAseq, with Illumina TruSeq library preparation and paired sequencing. Gene expression data were quantified as transcripts per million (TPM).

Changes in gene expression and platelet count were evaluated using the log_2_-based ratio of treated versus baseline readouts. The criteria for a low platelet count were defined as a count lower than 150,000 per μL. In addition, the log_2_ fold change between baseline and treated samples greater than 0.5 was used to exclude patients with a pre-existing thrombocytopenia condition at baseline.

The differences between vehicle control (preclinical study) or baseline (clinical trial) and treated groups were assessed for statistical significance using Analysis of Variance (ANOVA) followed by Dunnett’s multiple comparison test. An alpha threshold of 0.05 was employed, corresponding to a 95% confidence interval. Correlation analysis of gene expression changes was conducted using Pearson correlation coefficients. All statistical analyses were performed utilizing standard packages from the R statistical programming language (RRID: SCR_001905) and GraphPad Prism software (GraphPad Prism, RRID: SCR_002798).

## 3 Results

### 3.1 Confirmation of target engagement

To confirm target engagement, HEXIM1 was utilized as a pharmacodynamic marker for BET inhibition. The activation of HEXIM1 depends on the release of free positive transcription elongation factor b (pTEFb) following BET inhibition. Consistent upregulation of HEXIM1 with BET inhibitor treatment has been reported in previous studies (28–30). This confirmation of target engagement was conducted in both preclinical and clinical studies as part of exploratory research to identify biomarkers from preclinical to clinical settings.

**Figure 1:**
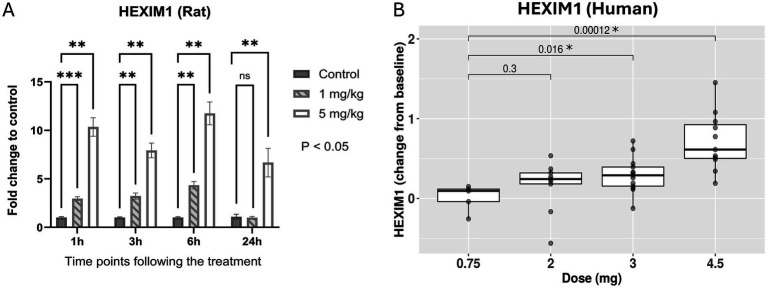
Change in HEXM1 expression upon BMS-986158 treatment in the rat and clinical trial patients. **(A)** Expression levels of HEXIM1 in rat blood at various time points following BMS-986158 treatment. The data are presented as fold changes relative to the vehicle control group. Asterisks indicate statistical significance (*p* < 0.05). **(B)** Expression levels of HEXIM1 in human patient blood during cycle 2 treatment at different doses. The X-axis represents the treatment dose, while the Y-axis shows the Log_2_ fold change between on-treatment and baseline levels for each individual patient. *p*-values are indicated with brackets, showing the levels of significant differences among treatment groups.

The preclinical data demonstrated a dose-dependent upregulation of HEXIM1, which was statistically significant at all time points of 1, 3, 6, and 24 h following 4-day daily treatments at doses of 0 (vehicle control), 1, and 5 mg/kg/day (Figure 1A). In the clinical study (NCT02419417), blood samples from all patients at the dose groups of 0.75, 1.25, 2.0, 3.0, or 4.5 mg were collected 8 h post the treatment on day 1 of cycle 2. The expression of HEXIM1 was statistically significant between the higher dose groups (3 and 4.5 mg) and the lowest dose group of 0.75 mg (Figure 1B). The target engagement of BMS-986158 treatment in rat and human clinical trials was confirmed by the dose-dependent upregulation of HEXIM1 observed in preclinical and clinical studies.

### 3.2 Reduction of platelets in blood and accumulation of megakaryocytes in bone marrow with BMS-986158 treatment

**Figure 2:**
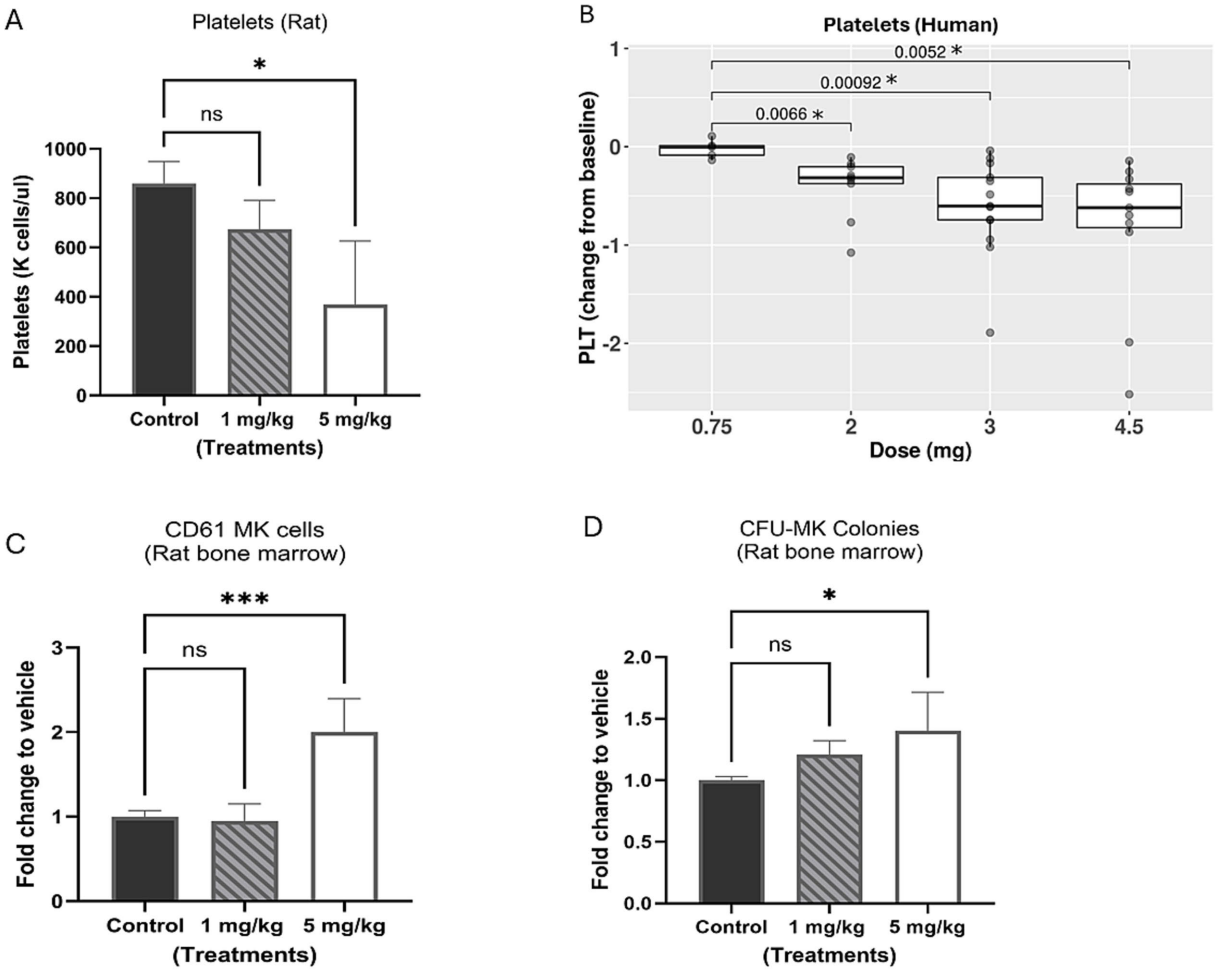
Evaluation of platelets in rat and clinical trial patient blood and megakaryocytes in rat bone marrow with BMS-986158 treatment. **(A)** Platelet counts in rat blood. **(B)** The platelet counts of clinical trial patients cross 3 schedules of all doses (0.75, 2, 3, and 4.5 mg) in the time range from Cycle 2 day 1 to day 23. **(C)** Megakaryocytes (CD61 positive cells) in rat bone marrow measured by flow cytometry. **(D)** The progenitors of megakaryocytes in rat bone marrow 5 days following the treatment of BMS-986158 at doses of 0 (vehicle control), 1, and 5 mg/kg /day for 4 days. X-axis: Treatment groups, Y-axis: platelet count, a number times a thousand per μl **(A)**, Log_2_ fold change of patient platelet counts from baseline to treatments **(B)**; fold change of treated groups to vehicle control **(C,D)**. Asterisks indicate statistical significance (*p* < 0.05). ns: no statistically significant difference.

The effect of BET inhibition on platelets in blood was assessed in a preclinical rat study and a human clinical trial (CA011-001; NCT02419417). In the rat study, the treatment doses of BMS-986158 were 0 (vehicle control), 1, and 5 mg/kg/day for 4 days, with selected doses below the lethal dose of 10 mg/kg/day tested in previous tolerability studies. Blood samples were collected for platelet count on day 5 post the completion of 4-day treatments. The results showed a dose-dependent reduction in platelets count, with the highest dose group of 5 mg/kg/day being statistically significant (*p* < 0.05) compared to the control group (Figure 2A).

In the human clinical trial, the reduction in platelet counts between the lowest dose group of 0.75 mg and the groups receiving 2, 3 and 4.5 mg of BMS-986158 treatments was statistically significant (Figure 2B). In this study, BMS-986158 was evaluated in patients with selected advanced solid tumors for a tolerable safety and dose-proportional pharmacokinetics (PK) profile. Although preliminary findings indicated that BMS-986158 monotherapy was tolerable, thrombocytopenia was observed in 39% of patients and was one of the most common treatment-related adverse events (TRAEs) (13, 31).

To evaluate the effect of BET inhibition on megakaryocytes in bone marrow, rat bone marrow cells were collected on day 5 post the treatment from the same rats from which blood samples were collected. The percentage of CD61-positive cells in the bone marrow showed significant increase of megakaryocytes at 5 mg/kg (Figure 2C). The bone marrow cells were also evaluated in the CFU assay to further assess the effect of BET inhibition on megakaryocytes (MK) progenitors. A dose-dependent increase of CFU-MK colonies was observed in the treated groups compared to the control group (Figure 2D).

The changes in platelet reduction in the blood and increased megakaryocyte progenitors in the bone marrow with the treatment doses were statistically significant from the control group. In summary, decreased platelets were observed in both preclinical and clinical studies, along with an accumulation of megakaryocytes in rat bone marrow, following treatment with BMS-986158.

### 3.3 Transcriptional expression impacted by BET inhibition, regulated by GATA1, and associated with thrombocytopenia

To identify appropriate and relevant biomarkers for predicting thrombocytopenia induced by BET inhibition, the mechanism of BET-inhibition-induced hematotoxicity through GATA1 regulation was investigated. Specifically, the effect of BMS-986158 on the transcriptional expression of GATA1, a transcription factor important in hematopoiesis, and its regulated genes involved in megakaryopoiesis and thrombopoiesis was evaluated. The NFE2 gene, expressed during erythropoiesis and megakaryopoiesis and essential for megakaryocyte maturation and platelet production, and PF4 gene, encoding a chemokine released by platelets associated with megakaryocytopoiesis (32, 33), were also evaluated using samples collected from both a preclinical study in rats and a clinical study (NCT02419417).

In a preclinical study, rats were dosed with vehicle control, 1 mg/kg/day, or 5 mg/kg/day of BMS-986158 for 4 days. Blood samples from five rats per group were collected at 1-, 3-, 6-, and 24-h post-treatment. Bone marrow cells were collected on day 5, 24 h after the 4-day treatment was completed. The gene expression levels of GATA1, PF4, and NFE2 were assessed in both blood and bone marrow samples.

**Figure 3:**
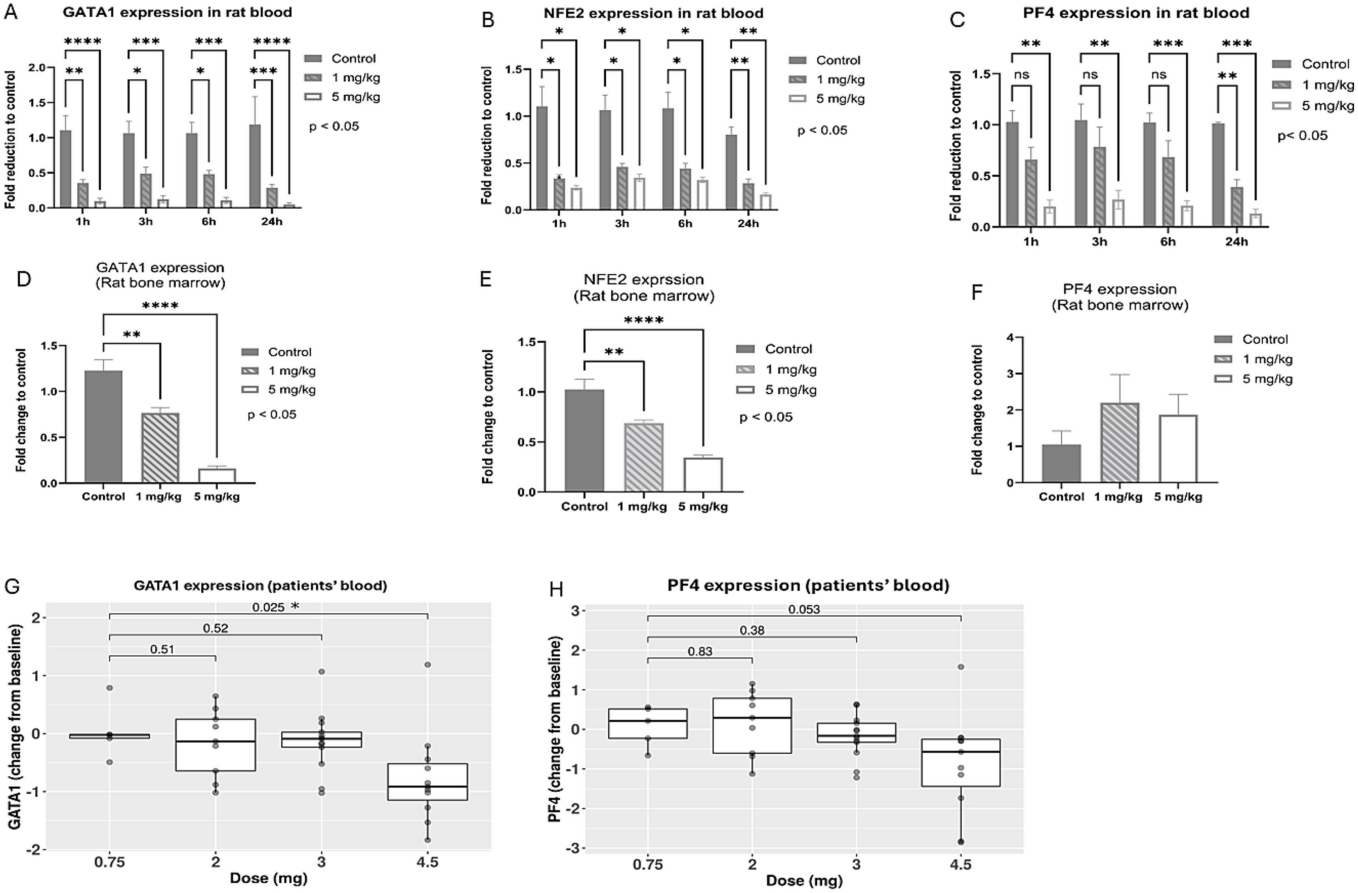
Expression levels of GATA-1 and associated genes, NFE2 and PF4, following BMS-986158 treatment in rats and clinical trial patients. Transcriptional expressions of GATA1 in rat blood **(A)** and bone marrow **(D)**, NFE2 in rat blood **(B)** and bone marrow **(E)**, PF4 in rat blood **(C)** and bone marrow **(F)** at 1, 3, 6, and 24 h post the treatment of BMS-986158 at doses of 0 (control), 1, and 5 mg/kg/day. Transcriptional expression changes of GATA1 **(G)** and PF4 **(H)** from baseline in patients treated with BMS-986158 at doses of 0.75, 2, 3, and 4.5 mg. The X-axis represents BMS-986158 treatment time points **(A–C)** and dose group **(D–H)**. The Y-axis shows transcriptional expression fold change relative to vehicle control **(A–F)**, and Log_2_ fold change from baseline **(G,H)**. *p* values in G and H are displayed with brackets. Asterisks indicate statistical significance (*p* < 0.05).

The results showed statistically significant, dose-dependent reductions in the transcriptional expressions of GATA1 and NFE2 in rat blood and bone marrow. These reductions were observed at all time points in the blood (Figures 3A,B) and within 24 h post-treatment in the bone marrow (Figures 3D,E). PF4 gene expression in rat blood also showed dose-dependent reduction, with the 5 mg/kg/day group exhibiting statistically significant reductions compared to the vehicle control at all time points. A time-dependent reduction in PF4 transcriptional expression level was observed, with statistically significant in the low dose group (1 mg/kg/day) at the 24-h time point (Figure 3C). However, in rat bone marrow, PF4 transcriptional expression increased with BMS-986158 treatment, although this change was not statistically significant (Figure 3F).

In the clinical study among all patients, the transcriptional expressions of GATA1 and PF4 exhibited dose-dependent downregulation (Figures 3G,H). GATA1 showed a statistically significant change from baseline when comparing the lowest dose group (0.75 mg) to the highest dose group (4.5 mg).

**Figure 4:**
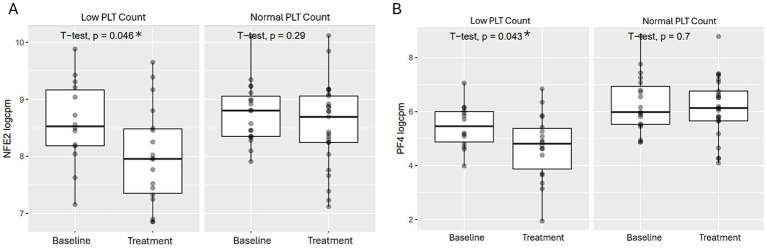
Comparison of GATA-1 regulated transcriptional expressions post BMS-986158 treatment in patients with low vs. normal platelet counts. Transcriptional expressions of NFE2 **(A)** and PF4 **(B)** in patient blood samples with either low or normal platelet counts. Clinical trial patient platelet counts cross 3 schedules of all doses (0.75, 2, 3, and 4.5 mg) in the time range from Cycle 2 day 1 to day 23. The criteria for low platelet count are described in the methods. The X-axis represents sample groups from either baseline or treated groups. The Y-axis shows Log_2_ gene transcript counts per million. Asterisks indicate statistical significance (p < 0.05).

The data also revealed that patients with thrombocytopenia (defined as < 150×10^3^/μl and > 0.5 Log_2_ fold change in platelet count from baseline) had significantly greater NFE2 and PF4 genes downregulation compared to those with normal platelet counts (Figures 4A,B).

The results from both preclinical and clinical studies demonstrated that BMS-986158 treatment downregulated GATA1, along with its regulated genes NFE2 and PF4 in blood, which are associated with thrombopoiesis.

### 3.4 Correlation between GATA1 expression and expression of NFE2 and PF4 genes with BMS-986158 treatment

Following the observed downregulation of GATA1, NFE2, and PF4 genes after BMS-986158 treatment in both rat and human studies, further investigation was conducted to determine whether the changes in PF4 and NFE2 transcriptional expression were associated with changes in GATA1 transcriptional expression in blood from rats or patients treated with BMS-986158.

Rat blood samples that showed statistically significant transcriptional changes of NFE2 and PF4 with BMS-986158 treatment were used for the correlation analysis. In the correlation analysis between GATA1 and NFE2, all treatment groups at all time points were included. For the correlation between GATA1 and PF4, only samples showing statistically significant transcriptional changes at 24 h post-treatment were used for the analysis.

**Figure 5:**
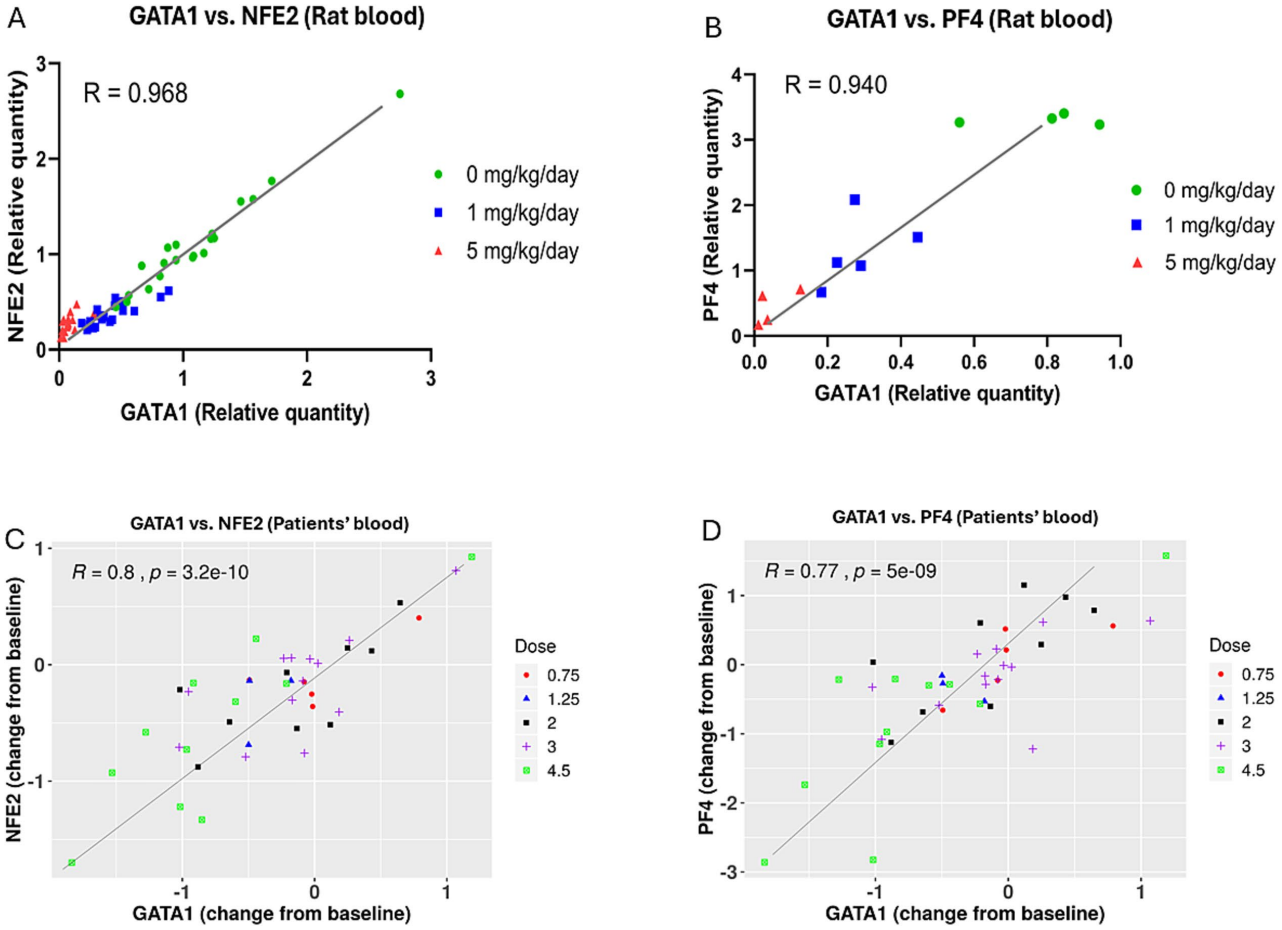
Correlation between GATA1 transcriptional expression and the transcriptional expressions of NFE2 and PF4 genes in blood from rats and patients treated with BMS-986158. **(A)** Correlation analysis between GATA1 and NFE2 in rat blood including all dose groups at all time points. **(B)** Correlation analysis between GATA1 and PF4 in rat blood at 24-h time point, including all dose groups. **(C)** Correlation analysis between GATA1 and NFE2 in clinical trial patient blood samples across three treatment schedules and five dose groups (0.75, 1.25, 2, 3, and 4.5 mg). **(D)** Correlation analysis between GATA1 and PF4 in clinical trial patient blood samples across the same treatment schedules and dose groups. The X-axis represents the relative quantity of the GATA1 gene expression, normalized to the vehicle control group in rats **(A,B)** or change from baseline in human blood **(C,D)**. The Y-axis shows the relative quantity of the NFE2 **(A)** and PF4 **(B)** genes expression, normalized to the vehicle control group in rats, or the Log_2_ fold change from baseline of the NFE2 **(C)** and PF4 **(D)** genes in human blood.

In rats, there was a high correlation between GATA1 and NFE2 transcriptional changes, with a correlation coefficient of R = 0.97 (Figure 5A), and between GATA1 and PF4 transcriptional changes, with a correlation coefficient of R = 0.94 (Figure 5B).

In clinical trial patients, there were good correlations between GATA1 and NFE2, with a correlation coefficient of R = 0.8, and between GATA1 and PF4, with a correlation coefficient of R = 0.77 (Figures 5C,D). These findings were based on blood samples collected from three schedules and five doses of BMS-986158 at 0.75, 1.25, 2, 3, and 4.5 mg.

### 3.5 Timing of GATA1, NFE2, and PF4 transcriptional expression change in relation to thrombocytopenia onset during BMS-986158 treatment

To evaluate the timing of transcriptional expression changes of GATA1, NFE2, and PF4 in relation to the onset of thrombocytopenia during treatment with BMS-986158, Table 2 summarizes the results from a phase 1/2a clinical study (NCT02419417).

**Table 2:** Timing of thrombocytopenia onset in relation to RNAseq analysis timing.

Treatment schedule	Time points of RNAseq analysis	Thrombocytopenia onset after the 1st dose in cycle 2	Percentage of patients with thrombocytopenia onset among total patients
Schedule A Total 9 cycles	Day 1 after the 1st Dose in Cycle 2	Day 8	4%
		Day 15 - day 21	48%
		Day 22 - day 30	48%
Schedule B Total 24 cycles	Day 14 after the 1st dose in Cycle 2	Day 15	20%
		Day 21	80%
Schedule C Total 19 cycles	Day 7 after the 1st dose in Cycle 2	Day 8	9%
		Day 14 - day 17	91%

The study included three schedules (A, B, and C), with treatment cycles ranging from 9 (Schedule A) to 24 (Schedule B) and 19 (Schedule C). Blood samples for RNAseq analysis were collected within treatment Cycle 2: at the beginning of the cycle, 8 h after the first dose on day 1 (Schedule A), at the end of Cycle 2 on day 14 (Schedule B), or on day 7 (Schedule C).

In Schedule A, only 4% of patients had documented thrombocytopenia on day 8, while the remaining 96% experienced it between days 15 and 30 and the RNAseq analysis was conducted on day 1 after the first dose in Cycle 2. In Schedule B, 20% of patients had thrombocytopenia by day 15, and 80% by day 21. The RNAseq analysis was conducted on day 14 after the first dose in Cycle 2. In Schedule C, only 9% of patients had thrombocytopenia on day 8, while 91% experienced it between days 14 and 17. The RNAseq analysis was conducted on day 7 after the first dose in Cycle 2. In summary, thrombocytopenia mostly occurred 2–3 weeks after the downregulation of NFE2 and PF4 gene expressions were measured and before subsequent treatment cycles after Cycle 2.

## 4 Discussion

BET inhibitors are being developed as a therapy for cancer due to the frequent dysregulation of BET proteins in solid tumors and leukemia, which results in the aberrant expression of oncogenes. Although partial responses have been observed in patients treated with BET inhibitors for cancers over the past decade, drug resistance and toxicity, particularly thrombocytopenia, remain significant challenges (13), and these issues have hindered full exploration of efficacy. Utilizing biomarkers to predict which patients are more likely to develop thrombocytopenia could be an effective approach to help explore a broader dose range for efficacy, where doses and schedules could be adjusted accordingly for those patients at risk for thrombocytopenia.

To identify biomarkers associated with BET inhibition induced thrombocytopenia, we initially conducted transcriptional profiling of GATA1, which showed significant (*p* < 0.05) decreased expression in blood from patients treated with 4.5 mg BMS-986158. GATA1, a transcription factor involved in hematopoiesis, drives the differentiation and maturation of erythroid cells and megakaryocytes (34, 35). BET family proteins are essential for full GATA1 activity (34). Mutations in GATA1 have been reported to result in thrombocytopenia (36). In the absence of functional BET proteins, GATA1 cannot effectively bind to its target sites and promote their transcriptional activation, leading to inefficient transcriptional activation or downregulation of the genes necessary for megakaryocyte maturation and platelet production (34). The downregulation of GATA1 expression results in impaired megakaryopoiesis, causing thrombocytopenia (37).

Based on this understanding, we investigated RNAseq profiles from patients treated with BMS-986158 to identify deregulated genes involved in megakaryocyte production and regulated by GATA1. Our study identified two genes, NFE2 and PF4, which showed significant transcriptional decrease following BMS-986158 treatment.

NFE2 is a transcription factor that works closely with GATA1. Both GATA1 and NFE2 co-localize at regulatory sites within the genome, coordinating the transcription of genes essential for erythroid differentiation and platelet production (38–40). In megakaryocytes, GATA1 and NFE2 are critical to produce platelets. GATA1 regulates genes for megakaryocyte maturation, while NFE2 regulates genes that control platelet production and function (38, 41). A deficiency in NFE2 can lead to severe thrombocytopenia in mammals, resulting in bleeding disorders due to impaired platelet production (42). In zebrafish, NFE2 is required for adult thrombocyte formation and function, indicating its crucial role in the later stages of thrombopoiesis (42). Therefore, NFE2 is crucial for the final stages of megakaryocyte maturation and platelet release (41).

GATA1’s regulation of PF4 is essential for the maturation of megakaryocytes. GATA1 binds to the promoter regions of the PF4 gene, activating its transcription in megakaryocytes. This regulation ensures that PF4 is produced at the right levels to support proper platelet function and development (39). Without adequate GATA1 activity, PF4 expression is reduced, leading to impaired megakaryocyte development and subsequent thrombocytopenia (39). The proper expression of PF4, driven by GATA1, is necessary for maintaining platelet function and preventing bleeding disorders. Deficiencies in GATA1, whether due to mutations or inhibition, can disrupt PF4 expression, contributing to platelet dysfunction and increased bleeding risk (39, 43). Therefore, GATA1 is crucial for the transcriptional activation of PF4, which in turn supports megakaryocyte maturation and platelet production.

The correlation analysis between GATA1 expression and the expression of NFE2 and PF4 genes revealed a strong correlation in both preclinical and clinical studies (Figure 5). This result further demonstrates the regulatory relationship of GATA1 with NFE2 and PF4, establishing NFE2 and PF4 as suitable biomarkers for predicting BMS-986158 induced thrombocytopenia. To enhance the predictive power of potential biomarkers for future treatment options, blood samples from all three treatment schedules with different time points were included, broadening the range of treatment schedules and increasing the sample size.

It has also been reported that the mutations in GATA1, such as those producing the truncated isoform GATA1s, can alter chromatin remodeling and gene expression in megakaryocytes (44). This leads to an early lineage bias toward megakaryocyte production but with incomplete differentiation, resulting in the accumulation of megakaryocyte progenitors (44). These changes in chromatin accessibility and long-range genomic interactions disrupt the normal megakaryopoiesis process, further contributing to the accumulation of immature megakaryocytes in the bone marrow (44). These previous findings explain the results from our rat bone marrow evaluation, which showed an increased percentage of megakaryocytes (Figure 2C), the colonies of megakaryocyte progenitors (Figure 2D), as well as higher expression of PF4 (Figure 3F) in the bone marrow with BMS-986158 treatment. Overall, the loss or deficiency of GATA1 disrupts the normal differentiation and maturation of megakaryocytes, leading to their accumulation in the bone marrow and associated hematopoietic abnormalities.

Our study demonstrates that BET inhibition induces thrombocytopenia *in vivo* by altering GATA1 gene expression and its downstream genes, NFE2 and PF4, which regulate megakaryopoiesis and thrombopoiesis. These findings identify NFE2 and PF4 as potential biomarkers for thrombocytopenia.

This finding aligns with clinical observations for other BET inhibitors such as Pelabresib, where thrombocytopenia is a prominent, dose dependent, and reversible side effect (45). Similarly, although Selinexor targets a different upstream pathway, it too has been associated with thrombocytopenia (46), indicating that dysregulation of platelet-related genes may be a common early indicator of pharmacology across converging upstream pathway interventional targets for diverse anticancer agents.

Further investigation into the use of NFE2 and PF4 as early biomarkers could improve our ability both to predict and manage drug induced thrombocytopenia.

The early detectability of gene changes within 24 h posts treatment underscores their potential for early prediction of thrombocytopenia. It is important to note that while gene expression changes in NFE2 and PF4 were observed at early time points in Schedule A (Day 1 after treatment), they were not substantial enough to reach statistical significance. Statistically significant changes were detected when later time points, specifically the end of Cycle 2 treatment—when sample data from Schedules B and C were incorporated. These findings suggest a lagging time between initial dosing and a significant transcriptional effect. This demonstrates the importance of selecting the appropriate day for sample collection based on peak transcriptional effect, notwithstanding or understanding if there could be a decrease in effect from peak.

Therefore, optimizing the timing of blood sample collection for biomarker assessment is critical. The time points selected in this study should be refined and adjusted for future studies based on varying doses and treatment durations. This approach ensures adequate drug exposure to achieve both therapeutic efficacy and significant transcriptional changes while enabling early identification of thrombocytopenia risk. Additionally, the proposed biomarkers NFE2 and PF4 for predicting thrombocytopenia were identified based on gene expression associations and consistency with published literature supporting the functional involvement of these targets in thrombopoiesis, however without direct experimental validation to demonstrate causation. To confirm the causal role of these genes associated with thrombocytopenia, further functional studies will be necessary such as assessing the impact of GATA1 knockdown, overexpression of NFE2 and PF4, or conducting a NFE2/PF4 loss-of-function study.

The current data demonstrates a correlation between BET inhibition and the expression of PF4 and NFE2. These genes have been evaluated as potential biomarkers and early predictors of thrombocytopenia, potentially enabling preemptive therapeutic responses (e.g., dose reduction) before thrombocytopenia manifests. Given that thrombopoiesis is regulated by a highly redundant and interconnected pathway, it is unlikely that BET inhibition is solely responsible for platelet formation. Moreover, because these biomarkers function downstream in this pathway, they may also prove valuable for monitoring the effects of various upstream pharmaceutical interventions, such as JAK inhibitors.

In recent years, clinical studies have increasingly explored combination therapies with BET inhibitors for cancer treatment, addressing the challenges of drug resistance and toxicity associated with single agent BET inhibitor therapy. One promising multi-drug strategy involves combining BET inhibitors with JAK inhibitors to enhance therapeutic efficacy.

For instance, BMS-986158 has demonstrated significant benefits in combination with ruxolitinib or fedratinib in treating myelofibrosis, with patients experiencing improved responses over time (47). However, the most common adverse effects observed in these clinical studies are thrombocytopenia and anemia (47). Furthermore, there has been increased interest in combining BET and JAK inhibitor treatment for myeloproliferative neoplasms (MPN) as JAK/BET inhibition results in a marked reduction in serum inflammatory cytokines, reduced disease burden and reversed bone marrow fibrosis *in vivo* in mouse MPN models (48).

Research has shown that JAK2 plays a significant role in regulating NFE2 expression. Mutations in JAK2, such as JAK2 V617F, lead to enhanced JAK/STAT activation, contributing to dysregulated gene expression, including the overexpression of NFE2. Another study highlights that JAK2, along with the histone demethylase JMJD1C, promotes NFE2 overexpression through epigenetic mechanisms (49). Since NFE2 is an important regulator in hematopoiesis, it plays a crucial role in red blood cell production. Mutations or dysregulation of NFE2 can impair erythropoiesis, leading to anemia (50). This suggests that NFE2 may serve as a biomarker not only for thrombocytopenia but also for anemia, extending its relevance beyond BET inhibition.

Finally, the consistent downregulation of NFE2 and PF4 transcription within hours post BMS-986158 treatment in both preclinical and clinical studies (Figures 3, 4), along with the observed dose-dependent decrease in platelet counts (Figure 2), supports their potential as prodromal biomarkers for predicting thrombocytopenia induced by BET inhibition. These results highlight their utility in early patient monitoring and management. Furthermore, our findings demonstrate the translatability of NFE2 and PF4 from preclinical to clinical settings, making them valuable tools for predicting and monitoring thrombocytopenia, and potentially anemia.

## Data Availability

The dataset has been published and archived at the European Genome-Phenome Archive (EGA). URL: https://ega-archive.org/studies/EGAS50000001162.

## Footnotes

^1^Source: https://clinicaltrials.gov
