# Supplementary figures and images for: NFE2 and PF4 as biomarkers for BET inhibition-induced thrombocytopenia in preclinical and clinical studies
Source: Front Med (Lausanne). 2025 Aug 27;12:1592693. doi: 10.3389/fmed.2025.1592693 (PMC12421914; doi:10.3389/fmed.2025.1592693)

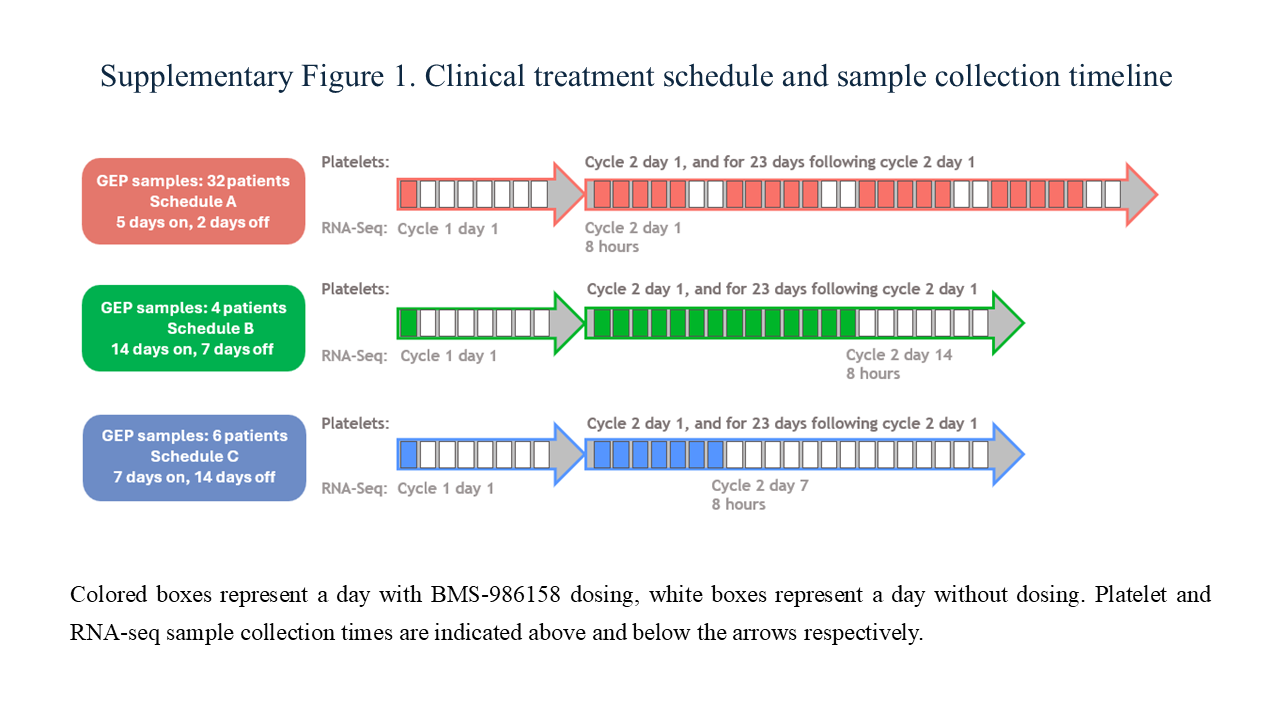

Supplement: Supplementary file 1 [file Image_1.tif]
